# The Atlanta Medical Society

**Published:** 1883-02

**Authors:** 


### Societies.

---

### THE ATLANTA MEDICAL SOCIETY.

*Reported for the Atlanta Medical Register.*

ATLANTA, GA., December, 1882.

The President, Dr. W. S. Armstrong, in the chair.

Dr. W. D. Bizzell read an essay on "The Contagiousness of Tuberculosis."

In the course of the subsequent discussion Dr. James B. Baird said he had listened with great interest to the exceedingly interesting and instructive review of this important subject which the essayist had so ably presented. During the reading of the suggestive paper one point after another that had occurred to him, as affording a basis for discussion, had been so fully considered by the speaker before he finished as to leave little for others to add.

The question of the contagiousness of phthisis pulmonalis is one of great practical importance and concern, which almost daily confronts us in our professional relationship, and in view of its far-reaching significance and most serious nature should be met in a practical and common sense manner. He thought that the history of this subject—notwithstanding the instances cited by the essayist, and others equally striking familiar to us all, which would seem to favor the contagious theory—sustained the negative view.

He confesses, however, to a predisposition, a bias, in favor of this solution of the problem, for the unavoidable suffering incident to the affection is ample without supplementing it by the distressing reflection that the unhappy viction may be the means of infecting his most devoted attendants, or his dearest relatives. It is a duty that other members of a family plainly owe to the afflicted member to give such aid and comfort as lies in their power. Yet many timorous persons would find the discharge of this duty, even if prompted by an ardent affection, a very great trial if the probability of contracting the fatal disease was decided to be a contingency. Many physiciaus express themselves recklessly and apparently thoughtlessly on this very point, and he has known haphazard opinions lightly proclaimed to occasion much anxiety and much genuine grief. Consumption is bad enough at best and it is cruel to increase the burden by laying on hypothetical ills. Let us for these reasons, if for no others, be slow to accept new doctrines which teach the contagiousness of pulmonary tuberculosis. The experiments on animals which have been relied upon to sustain this theory should be received with a liberal allowance. The subjects of these experiments are always under artificial, unnatural, influences. The surroundings of these animals are such as tend to impair or destroy their nutritive activity and to invite tuberculosis. The results observed by experimenters are, no doubt, often due solely to this cause and happen regardless of the experimental exposure. Monkeys, confined in cages, afford a striking illustration of this fact, for most of them, as is well known, die of consumption.

As to the existence of the so-called bacillus of tubucerlosis, described by several German investigators, notably by Koch, he is exceedingly sceptical. No doubt bacteria are there, but are they the cause of the disease or the result of the retrograde metamorphosis or the putrid decomposition which may also be present? This German bug business, he thinks, is being overdone.

Consumption, he regards as a failure of nutrition Whether the fault lies primarily in the nervous system—in the so-called trophic cells—in deficient or perverted action of the absorbent system, in mal assimilation or defective elective affinity on the part of the tissues for suitable nutrient material, or some where else, he cannot undertake to say, and perhaps the practical importance of its decision is not great. So far as the treatment of this disease is concerned he thinks he can safely say that success depends upon our power to promote nutrition. Just in proportion as we are successful in this will we achieve satisfactory results, but if we fail here the progress of the patient will be downward and the inevitable termination will soon draw near. The disease is defective nutrition. The cure must be improved, restored nutrition.

Dr. W. S. Armstrong reported the following case which has been under his charge for eight weeks. On the night of September 25th, a police officer of this city received a stab from a prisoner whom he had arrested and was conveying to prison. The wound was inflicted by a knife at the junction of the middle and upper third of the thigh, dividing the muscles, transversely, on the front of the thigh. The gash was about four inches in length, and extended to the femur. The femoral artery was missed about one inch.

After the hemorrhage had ceased the edges of the wound were drawn together by sutures and adhesive strips.

On the fourth day the sutures and dressings were removed. The parts seemed to have united. The pulse was good, the appetite fair. On the seventh night he awoke with a cramp in the whole leg, followed by pain in the wound, accompanied by a slight oozing of blood at the external extremity of the wound.

He was let alone, but three days later he experienced pain and febrile excitement. A probe was inserted into the wound and some adhesions torn up. Dark blood, in small quantity, slightly offensive, issued. The wound was then washed out with a three or four per cent. solution of carbolic acid and dressed with carbolized oil. The fever continued until October 10. On that day, at ten o'clock A.M., he was seized with a chill, severe pain in the lower part of the thorax, shoulder and neck on the left side Blood-poisoning was suspected. This condition developed into an attack of pneumonia involving the whole of the left lung. The sputa was mostly dark blood. The patient was very ill.

October 19th the improvement was great, and appetite good, but on the 25th he had a rigor with pain in the wounded limb from the groin to the foot. The whole limb was congested, presented a bluish color, and swelled rapidly.

He regarded it as phlebitis. The circulation was impeded and effusion had taken place within the areolar tissue, causing the limb to swell to twice its natural size. Petechiæ the size of a pea studded its surface. The pulse was very rapid, and death was apparently near. The swelling and discoloration of the integument extended over the abdomen and to the crest of the ilium.

On 28th he rallied, his appearance improved and some doubt as to the character of the effusion was felt. On 30th elevated the foot and made two punctures, two to four inches above and below the wound. Serum and some bloody fluid poured out for several days. The size of the limb was reduced.

Improvement progressed until November 8. Then a slight rise in pulse was noted. It ranged from eighty to one hundred.

On November 9th, complained of pain in the posterior part of the right side. Discharged rusty sputa. Had pneumonia affecting the right lung. Symptoms not so severe as in first attack. On 14th symptoms subsided, and to-day, 18th, he is doing well and sitting up.

These conditions—pneumonia, phlebitis, etc., were the result of septicæmia. The muscular cramp pulled apart the freshly united wound, blood was effused, it decomposed, and blood poisoning resulted. While the man is progressing satisfactorily to-day, unfavorable symptoms may arise at any time.

The treatment consisted in the use of tonics—quinine, iron, etc., with whisky or brandy, and a generous, digestible diet. The wound has been entirely healed for several weeks. There was very little suppuration from the beginning to the end.

Most of the time his temperature was little above normal. It was never very high. The day after the phlebitis developed the pulse was 140. Temperature, under the tongue, 97.5°. It was once 103°. The patient presented during the phlebitis a cadaverous, sunken appearance, and it was the opinion of several physicians, who saw him at the time, he would not live thirty-six hours.

NOTE.—January 21, 1883. His condition continued to improve and he was able to walk about the house and to ride out. To all appearances he had recovered from the effects of blood poisoning. But on the 10th of January 1883, he experienced pain in the left leg from the knee to the foot, which was followed by pain and marked swelling in the region of the calf. These symptoms had nearly passed away on the 17th when he was attacked with severe pain in the right side which developed into pneumonia involving the whole lung posteriorly, and from which he is suffering at this date.

